# Analyzing Patient Experience on Weibo: Machine Learning Approach to Topic Modeling and Sentiment Analysis

**DOI:** 10.2196/59249

**Published:** 2024-11-29

**Authors:** Xiao Chen, Zhiyun Shen, Tingyu Guan, Yuchen Tao, Yichen Kang, Yuxia Zhang

**Affiliations:** 1 Department of Nursing, Fudan University Zhongshan Hospital, Room 501, No 180 Fenglin road, Xuhui District, Shanghai, 200032, China, 86 13816881925, 86 64041990

**Keywords:** patient experience, experience, attitude, opinion, perception, perspective, machine learning, natural language process, NLP, social media, free-text, unstructured, Weibo, spatiotemporal, topic modeling, sentiment

## Abstract

**Background:**

Social media platforms allow individuals to openly gather, communicate, and share information about their interactions with health care services, becoming an essential supplemental means of understanding patient experience.

**Objective:**

We aimed to identify common discussion topics related to health care experience from the public’s perspective and to determine areas of concern from patients’ perspectives that health care providers should act on.

**Methods:**

This study conducted a spatiotemporal analysis of the volume, sentiment, and topic of patient experience–related posts on the Weibo platform developed by Sina Corporation. We applied a supervised machine learning approach including human annotation and machine learning–based models for topic modeling and sentiment analysis of the public discourse. A multiclassifier voting method based on logistic regression, multinomial naïve Bayes, and random forest was used.

**Results:**

A total of 4008 posts were manually classified into patient experience topics. A patient experience theme framework was developed. The accuracy, precision, recall, and F-measure of the method integrating logistic regression, multinomial naïve Bayes, and random forest for patient experience themes were 0.93, 0.95, 0.80, 0.77, and 0.84, respectively, indicating a satisfactory prediction. The sentiment analysis revealed that negative sentiment posts constituted the highest proportion (3319/4008, 82.81%). Twenty patient experience themes were discussed on the social media platform. The majority of the posts described the interpersonal aspects of care (2947/4008, 73.53%); the five most frequently discussed topics were “health care professionals’ attitude,” “access to care,” “communication, information, and education,” “technical competence,” and “efficacy of treatment.”

**Conclusions:**

Hospital administrators and clinicians should consider the value of social media and pay attention to what patients and their family members are communicating on social media. To increase the utility of these data, a machine learning algorithm can be used for topic modeling. The results of this study highlighted the interpersonal and functional aspects of care, especially the interpersonal aspects, which are often the “moment of truth” during a service encounter in which patients make a critical evaluation of hospital services.

## Introduction

Understanding patient experience is central to improving care delivery, and structured questionnaires capturing what actually happened to patients during a hospital stay have been widely used to provide insight into health care quality and derive priorities for quality improvement. In addition to hospital-initiated quantitative data, several types of patient experience feedback are currently available [1], and patient‐initiated qualitative feedback posted online, such as complaints or comments on social media, has gained increasing popularity because of its potential for identifying patients’ in-depth concerns.

Worldwide, social media platforms have an increasing number of users, and social media users can reach a wide audience quickly and efficiently [2,3]. Social media has become an indispensable platform for expressing opinions among the public. Given the importance of health, it is not surprising that people provide opinions and comments on hospitals and their health care experience on social media platforms [4]. More importantly, a recent study showed that patient experience was readily elicited through qualitative analysis of social media posts rather than through conventional interviews [5]. Owing to the benefits of accessibility, flexibility, and anonymity, social media has become a ubiquitous tool that allows individuals to openly gather, communicate, and share real-time feedback about their interactions with health care services [6]. Comments and posts on social media platforms can be especially valuable for health care quality improvement if they are analyzed scientifically and efficiently.

The rapid advancement of data mining techniques offers a potential opportunity for using the abundant online free-text comments and posts regarding health care experience [7], and further analyzing the data-rich information contained within patients’ online patient feedback may be a promising way to understand patient experience, deduce public attitudes, and facilitate quality improvement, supplementing traditional survey approaches [8]. Several studies [9,10] have used natural language processing to extract meaningful information from free-text patient feedback data, identify the sentiment conveyed by patients, and further highlight areas of concern from patients’ perspectives from which health care providers should act. Moreover, this approach could provide essential information that is unavailable in conventional quantitative surveys [11]. Since patients are familiar with social media platforms, they are inclined to use these platforms as more trusted channels for freely expressing their authentic feelings and providing information about what they truly value in their experiences [12]. Patients’ free-text feedback could also predict their survey-based rating of health care services. Greaves et al [13] applied a machine learning classification approach to group unstructured online comments regarding health care experience into categories and analyzed their associations with traditional patient experience surveys; their results revealed that free-text feedback could predict patients’ quantitative ratings of hospital care with reasonable accuracy.

As the world becomes more digitally oriented, social media platforms will be an increasingly important channel for health care promotion. The public discourse on social media platforms is relatively long, and the patient experience data are always cast into a multilabel classification problem [7,14]. However, although patients’ discourse always mentions more than one discussion topic, most previous topic modeling studies tended to label patient comments with one category [6,7], resulting in the underestimation of patients’ discussion topics. Moreover, applying data mining techniques to analyze the content of comments in social networks remains a nascent technology in the health care industry, and studies on the use of social media posts to capture patient experience have focused mainly on English-speaking users. Little is known about what the Chinese public discusses during their encounters with hospitals on Chinese social media platforms.

In 2020, China achieved an internet penetration rate of 67%, with approximately 940 million individuals using the internet [15]. Sina-Weibo (Sina Corporation), known as the Chinese version of Twitter, is the most popular social media platform in China; public opinions expressed on Weibo always reveal existing social concerns and issues in China. To address the gap in the literature, we used machine learning techniques, including sentiment analysis and topic modeling, to conduct spatial-temporal analysis of the user-generated content on Weibo to identify key issues or topics related to the health care experience and determine which aspects of health care services have the most important impact on patient satisfaction. To our knowledge, this study is the first to examine the public discourse on patient experience among Chinese social media users. The findings provide a deeper understanding of patient experience and actionable insights for health care organizations and professionals to improve quality.

Therefore, this study aimed to identify common discussion topics related to health care experience from the public’s perspective and to determine areas of concern from patients’ perspective on which health care providers should act.

## Methods

### Study Design

**Figure 1.**
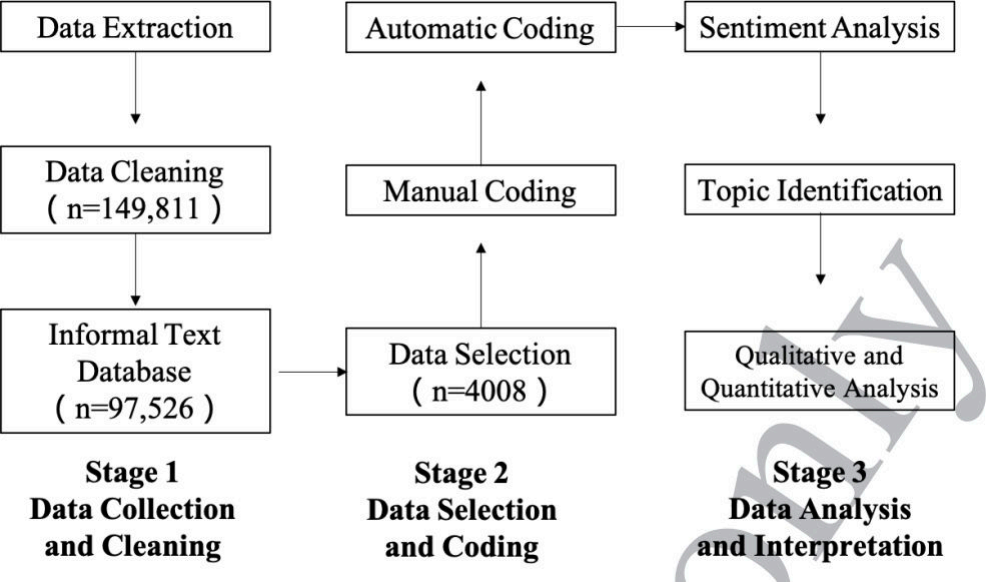
Study design.

We conducted spatiotemporal analysis of the volume, sentiment, and topic of patient experience–related posts on the Weibo platform. Figure 1 shows an overview of this study, which included three distinct stages: (1) data collection and cleaning, (2) data selection and coding, and (3) data analysis and interpretation.

### What This Study Adds

First, this study shows that the integration of logistic regression (LR), multinomial naïve Bayes (MNB), and random forest (RF) yields the highest prediction power, and the performance is better than that of any single one machine learning model.

Second, the results of sentiment analysis show that people tend to write negative posts on social media platforms, which could attract more responses and trigger more discussions.

Third, the results of topic modeling highlight the interpersonal and functional aspects of care, especially the interpersonal aspects, which are often the “moments of truth” during a service encounter in which patients make a critical evaluation of hospital services.

### Data Collection and Preprocessing

Weibo is the most widely used social media platform in China, with more than 550 million monthly active users [15], on which people publicly express their feelings and attitudes. We therefore selected the Weibo platform as the source to systematically search for posts about patient experience, defined as patients,’ friends,’ or family members’ discussions of hospital experience. Weibo supports the retrieval of textual content from a defined period that contains specific keywords. Weibo posts are open and easily accessible to everyone, and we did not extract users’ identities, such as poster ID and name, to maintain the users’ anonymity and avoid any invasion of individuals’ privacy. Moreover, only the post content, release date, and location were extracted to prevent identification of the users. Thus, there were no ethical issues to address.

To improve the search efficiency, we used a public free Python (Python Software Foundation) crawler tool to search for posts related to patient experience that contained predefined keywords. Hospital service–related terms and experience-related terms were combined to construct the final search strategy. Hospital service–related terms included hospital, health care service, doctor, nurse, and medical staff. With respect to experience-related terms, both broad and specific search terms were used to minimize the loss of relevant posts; broad search terms included experience, satisfaction, good, terrible, poor, and quality; specific search terms were determined on the basis of the 2012 National Health Service (NHS) patient experience framework, and terms including respect, environment, technology, information, education, communication, continuity, waiting time, trust, decision-making, access, emotional support, attitude, pain, and comfort were used. Patients’ opinions have time validity. To obtain representative patient voices, we searched for posts published in the last 10 years. Therefore, the release date of the posts was set from 2013 to 2023. We obtained 149,811 Weibo posts and stored the extracted posts locally in an informal text database. After some duplicate and reposted posts were filtered, 97,526 posts remained.

The content of posts on the Weibo platform is unstructured. For further processing and analysis, we preprocessed the text by removing garbled codes, hashtags, outliers, and non-Chinese characters and additional spaces.

### Data Selection

A manual method was used to identify relevant data. We set criteria including three stages for the inclusion and exclusion of retrieved posts for detailed analysis. First, posts were included if they covered any information about health care. We then estimated whether the post was released from the perspective of patients or their caregivers, and posts related to health-related knowledge, propaganda promotion from hospitals, and the working experience of medical staff or students were excluded. Finally, we determined whether patient experience was the main topic of the post; we excluded retrieved posts if they expressed only personal feelings of some specific illnesses.

XC and ZS selected relevant posts; if there was any disagreement, two other authors, YZ and Wei Qin, MD, were consulted, and a consensus was reached on whether the post should be included. Finally, 4008 valid posts were further analyzed.

### Data Coding

#### Manual Coding

Two researchers with expertise in patient experience independently coded 20% of the total posts to develop and refine the coding framework on the basis of the 2012 NHS patient experience framework [16] and the Chinese patient experience framework [17]. To ensure that the categories accurately represent the data, during the process of manual coding, the authors could also develop new codes if new content appeared in the articles. The final coding framework is shown in Multimedia Appendix 1. Each post was ascribed to one or more patient experience themes from the framework. In addition, these two researchers used the same dataset to perform sentiment analysis. According to the emotional attributes of the content, researchers determined and labeled each post with positive sentiment, negative sentiment, or mixed sentiment. If the content was a complaint, it was labeled with negative sentiment, whereas if the content was praise, it was labeled with positive sentiment. There are also some posts with mixed sentiment. For example, “the doctors treated me patiently, but the nurses were rude.” Multimedia Appendix 2 shows some specific examples of common themes. To ensure accuracy and fidelity with regard to the original meaning of the posts, we invited two doctoral nursing students with international study experience to independently translate the posts into English. With respect to the differences between the two translation texts, the research team traced the origin of the words and meanings in the English version to reach a consensus.

Manually coded posts were used as the learning template to predict the remaining posts via machine learning algorithms. To increase the credibility and dependability of the training dataset, the interrater agreement of each theme and sentiment was calculated to limit personal bias. The interrater agreement (Cohen κ) among the two annotators ranged from 0.81 to 0.93. In accordance with Landis and Koch [18], the threshold for substantial agreement was set at 0.61. During the process of coding, the authors could also develop new codes if new content appeared in the articles. If there was any disagreement, two other authors YZ and Wei Qin, MD, were consulted, and a consensus was reached on which theme the post fell into.

#### Machine Learning Modeling

Before machine learning modeling was used, we used the stop word tool to eliminate certain words that do not have any meaning in the text [19]. The patient experience data were cast into a multilabel classification problem. We used six distinct machine learning approaches, namely decision tree, support vector machine, LR, XGBoost, MNB, and RF, to predict patient experience posts. We divided the dataset into training or test datasets (70.1%/29.9%), which meant that the training dataset and test dataset have 562 and 240 pieces of posts, respectively. The prediction accuracy of the machine learning models was evaluated via the training dataset. According to the evaluation results, LR, MNB, and RF had better performance, with relatively high accuracy, precision, recall, and F-measure values (Tables1  2). To obtain the best performing model, we used a multiclassifier hard voting strategy to combine these three good-performing machine learning models to obtain the final classification results in the processing step [20]. As shown in Tables1  2, the performance metrics of multiclassifier collaborative tagging were excellent. We therefore integrated LR, MNB, and RF using hard voting to construct a classifier for predicting the discussion topics and sentiment of the remaining patient experience posts. Additionally, we used zero-shot learning to classify patient experience posts into five different emotion categories: happy, angry, sad, surprised, and afraid.

**Table 1.** Performance metrics of machine learning models for topic classification.

Model	Accuracy		Precision		Recall		F-measure	
	Training dataset	Testing dataset	Training dataset	Testing dataset	Training dataset	Testing dataset	Training dataset	Testing dataset
LR^a^	0.94	0.93	0.92	0.85	0.87	0.8	0.87	0.8
RF^b^	0.96	0.93	0.78	0.89	0.93	0.8	0.8	0.81
XGBoost	0.98	0.94	0.84	0.62	0.98	0.82	0.9	0.68
MNB^c^	0.94	0.93	0.99	0.95	0.74	0.73	0.84	0.82
SVM^d^	0.95	0.93	0.86	0.83	0.93	0.81	0.86	0.79
DT^e^	0.99	0.92	0.99	0.51	0.99	0.54	0.99	0.52
RF + LR + MNB	0.94	0.93	0.95	0.95	0.85	0.77	0.87	0.84

a LR: logistic regression.

b RF: random forest.

c MNB: multinomial naïve Bayes.

d SVM: support vector machine.

e DT: decision tree.

**Table 2.** Performance metrics of machine learning models on sentiment classification.

Model	Accuracy		Precision		Recall		F-measure	
	Training dataset	Testing dataset	Training dataset	Testing dataset	Training dataset	Testing dataset	Training dataset	Testing dataset
LR^a^	0.86	0.77	0.91	0.85	0.86	0.77	0.87	0.8
RF^b^	0.99	0.80	0.99	0.87	0.99	0.8	0.99	0.82
XGBoost	0.92	0.79	0.94	0.87	0.92	0.79	0.92	0.82
MNB^c^	0.81	0.76	0.92	0.91	0.81	0.76	0.84	0.81
SVM^d^	0.81	0.72	0.93	0.96	0.81	0.72	0.85	0.81
DT^e^	0.97	0.7	0.97	0.72	0.97	0.7	0.97	0.71
RF + LR + MNB	0.93	0.8	0.96	0.89	0.93	0.8	0.94	0.83

a LR: logistic regression.

b RF: random forest.

c MNB: multinomial naïve Bayes.

d SVM: support vector machine.

e DT: decision tree.

### Data Analysis

The statistical analysis was conducted via Python and IBM SPSS Statistics (version 26; IBM Corp). To efficiently extract and count each topic, all qualitative data were binarized to address multilabel classification via one hot encoding. The machine-coded data were subsequently imported into SPSS software to describe the characteristics of the discussion topics and sentiments of the posts and to calculate interrater agreement via Cohen κ values. Word clouds were created to visualize patient experience from frequently occurring topics in the posts [21].

### Patient and Public Involvement

Patients or the public were not involved in the design, conduct, reporting, or dissemination plans of this research.

### Ethical Considerations

This study was approved by the Research Ethics Committee of Zhongshan Hospital of Fudan University (approval B2022-613R). We did not extract users’ identities, such as poster ID and name. Moreover, only the post content, release date, and location were extracted to prevent identification of the users. Therefore, there is not any invasion of individuals’ privacy.

## Results

### Characteristics of the Included Patient Experience–Related Posts

A total of 4008 posts pertaining to patient experience–related text were identified. The average length of the posts was 257 (SD 241) words. Among these posts, 47.68% (n=1911) commented on outpatient departments, 24.63% (n=987) commented on inpatient departments, 6.81% (n=273) commented on emergency departments, 3.42% (n=137) commented on physical examination departments, and 19.31% (n=774) commented on hospital services and did not focus on a specific care setting. In terms of comment objects, 56.69% (2272/4008) described doctors, 49.15% (1970/4008) described nurses, 0.62% (25/4008) described health care assistants, 2.35% (94/4008) described nonhealth care workers, and 12.67% (508/4008) used the term “medical staff” without the particular objects.

**Figure 2.**
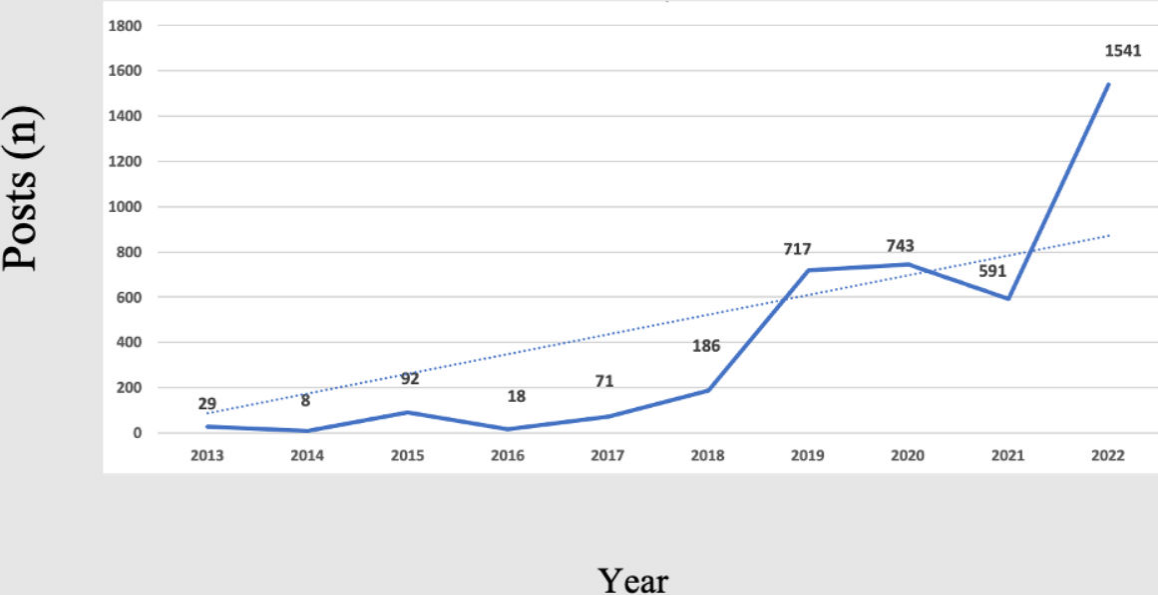
Frequency of posts by year.

The number of patient experience–related posts between 2003 and 2022 is illustrated in Figure 2. Despite occasional fluctuations, patient experience–related posts revealed a general trend of rapid increase. The peaks in 2020 and 2022 were contemporaneous with the outbreaks of COVID-19.

### Performance Metrics of Machine Learning Models

Tables1  2 list the performance metrics of the machine learning models. The accuracy, precision, recall, and F-measure of the integration of LR, MNB, and RF for patient experience themes were 0.93, 0.95, 0.80, 0.77, and 0.84, respectively. For the patient experience sentiment, the accuracy, precision, recall, and F-measure were 0.80, 0.89, 0.80, and 0.83, respectively.

### Sentiment Analysis

The sentiment analysis of the included Weibo posts revealed that negative sentiment posts constituted the highest proportion (3319/4008, 82.81%), followed by positive sentiment posts (635/4008, 15.84%), and then mixed sentiment posts (n=54, 1.35%). The word count of negative sentiment posts was significantly greater than that of positive posts (264 vs 229, *P*<.001). The results of zero-shot emotion recognition revealed that angry emotion constituted the highest proportion (1491/4008, 37.20%), followed by sad emotion (1029/4008, 25.67%), surprised emotion (647/4008, 16.14%), happy emotion (528/4008, 13.17%), and afraid emotion (313/4008, 7.81%). The outbreak of the COVID-19 pandemic was used as the cutoff value to divide this study’s period into the two time frames (2003‐2019 and 2020‐2023) to analyze the temporal aspects of Weibo post sentiment. From 2003‐2019, the percentage of negative sentiment posts was 89.74% (1006/1121), which was significantly greater than that from 2020‐2023, which was 80.12% (2313/2887). We further compared the sentiment distributions in different setting categories.

### Topic Modeling

Among the public discourse included in this content analysis, 3.89% (156/4008) of patient experience posts were nonspecifically about some specific care aspects and were labeled as “general.” Twenty patient experience themes were discussed on the social media platform (Table 3), and 52.74% (2114/4008) discussed more than one theme. The majority of the posts described the interpersonal aspects of care (73.53%, 2947/4008). The thematic distributions of the whole sample and of the user groups and sentiments are reported in Table 3. The theme distribution in the two time frames (2003‐2019 and 2020‐2023) is reported in Table 4.

The five most frequent themes were “health care professionals’ attitude,” “access to care,” “communication, information, and education,” “technical competence,” and “efficacy of treatment.” Multimedia Appendix 2 includes some specific examples of each theme.

**Table 3.** Patient experience themes associated with sentiment in different settings. Themes are ordered on the basis of their frequency in each hospital setting.

Order^a^	Outpatient department		Inpatient department		Emergency department	
	Negative (n=1588), n (%)	Positive (n=298), n (%)	Negative (n=795), n (%)	Positive (n=185), n (%)	Negative (n=248), n (%)	Positive (n=19), n (%)
1	Health care professionals’ attitude, 1048 (65.99)	Health care professionals’ attitude, 245 (82.2)	Health care professionals’ attitude, 501 (63)	Health care professionals’ attitude, 127 (68.6)	Health care professionals’ attitude, 163 (65.7)	Health care professionals’ attitude, 12 (63)
2	Access to care, 597 (37.59)	Information, communication, and education), 61 (20.5)	Information, communication, and education, 238 (29.9)	Information, communication, and education, 44 (23.8)	Access to care, 127 (51.2)	Access to care, 5 (26)
3	Information, communication, and education), 368 (23.17)	Technical competence, 47 (15.8)	Access to care, 154 (19.4)	Technical competence, 33 (17.8)	Information, communication, and education, 50 (20.2)	Emotional support, 3 (16)
4	Efficacy of treatment, 147 (9.26)	Access to care, 41 (13.8)	Efficacy of treatment, 73 (9.2)	Efficacy of treatment, 27 (14.6)	Efficacy of treatment, 12 (4.8)	Information, communication, and education, 3 (16)
5	Technical competence, 114 (7.18)	Emotional support, 22 (7.4)	Technical competence, 57 (7.2)	Responding requests, 15 (8.1)	Technical competence, 9 (3.6)	Efficacy of treatment, 2 (11)
6	Physical comfort, 52 (3.27)	Efficacy of treatment, 20 (6.7)	Responding requests, 39 (4.9)	Emotional support, 14 (7.6)	Responding requests, 7 (0.8)	Medical cost, 2 (11)
7	Responding requests, 49 (3.09)	Hospital environment, 17 (5.7)	Medical cost, 33 (4.2)	Hospital environment, 10 (5.4)	Hospital environment, 3 (1.2)	Responding requests, 2 (11)
8	Medical cost, 40 (2.52)	Responding requests, 16 (5.4)	Physical comfort, 23 (2.9)	Medical cost, 7 (3.8)	Continuity of care, 3 (1.2)	Hospital environment, 2 (11)
9	Hospital environment, 22 (1.39)	Physical comfort, 14 (4.7)	Involvement of family members, 19 (2.4)	Access to care, 6 (3.2)	Service process, 2 (0.8)	Service process, 2 (11)
10	Privacy, 21 (1.32)	Medical cost, 10 (3.4)	Hospital environment, 19 (2.4)	Physical comfort, 6 (3.2)	Emotional support, 2 (15.8)	Technical competence, 1 (5)
11	Service process, 19 (1.20)	Service process, 8 (2.7)	Error in treatment, 16 (2)	Sense of responsibility of staff, 6 (3.2)	Error in treatment, 1 (0.4)	Continuity of care, 1 (5)
12	Continuity of care, 19 (1.2)	Equipment, 8 (2.7)	Privacy, 14 (1.8)	Equipment, 5 (2.7)	Sense of responsibility of staff, 1 (0.4)	Food, 1 (5)
13	Equipment, 16 (1.01)	Sense of responsibility of staff, 2 (0.7)	Equipment, 12 (1.5)	Food, 3 (1.6)	Medical cost, 1 (0.4)	Equipment, 1 (5)
14	Excessive treatment, 13 (0.82)	Continuity of care, 1 (0.2)	Service process, 10 (1.3)	Service process, 3 (1.6)	—^b^	—
15	Emotional support, 12 (0.76)	Fairness of care, 1 (0.3)	Continuity of care, 10 (1.3)	Continuity of care, 2 (1.1)	—	—
16	Fairness of care, 11 (0.69)	Error in treatment, 1 (0.3)	Food, 9 (1.1)	Involvement of family members, 2 (1.1)	—	—
17	Sense of responsibility of staff, 7 (0.44)	Excessive treatment, 1 (0.3)	Excessive treatment, 5 (0.6)	Privacy, 1 (0.5)	—	—
18	Involvement of family members, 6 (0.38)	—	Sense of responsibility of staff, 4 (0.5)	—	—	—
19	—	—	Emotional support, 3 (0.4)	—	—	—
20	—	—	Fairness of care, 1 (0.1)	—	—	—

a Order is based on theme’s frequency in each hospital setting (1=most frequent; 20=least frequent).

b Not available.

**Table 4.** Changes in the patient experience themes in the Weibo posts over time.

Patient experience theme	Year 2013‐2019 (n=1121), n/N (%)	Year 2020‐2023 (n=2887), n/N (%)
Health care professionals’ attitude	784/1121 (69.94)	1928/2887 (66.78)
Access to care	342/1121 (30.51)	636/2887 (22.03)
Information, communication, and education	273/1121 (24.35)	616/2887 (21.34)
Technical competence	72/1121 (6.42)	321/2887 (11.12)
Efficacy of treatment	117/1121 (10.44)	215/2887 (7.45)
Responding request	27/1121 (2.41)	116/2887 (4.02)
Medical cost	23/1121 (2.05)	98/2887 (3.39)
Physical comfort	13/1121 (1.16)	104/2887 (3.6)
Hospital environment	27/1121 (2.41)	75/2887 (2.6)
Privacy	11/1121 (0.98)	52/2887 (1.8)
Emotional support	7/1121 (0.62)	53/2887 (1.84)
Equipment	8/1121 (0.71)	49/2887 (1.70)
Service process	6/1121 (0.54)	50/2887 (1.73)
Continuity of care	1/1121 (0.09)	41/2887 (1.42)
Error in treatment	8/1121 (0.71)	30/2887 (1.04)
Involvement of family members	5/1121 (0.45)	22/2887 (0.76)
Sense of responsibility of staff	0/1121 (0.00)	20/2887 (0.69)
Excessive treatment	7/1121 (0.62)	16/2887 (0.55)
Food	2/1121 (0.18)	14/2887 (0.48)
Fairness of care	2/1121 (0.18)	12/2887 (0.42)

### Health Care Professionals’ Attitude

The majority of patient experience–related posts (2712/4008, 67.66%) discussed health care professionals’ attitudes. People reported positive feelings and high satisfaction levels when they found health care providers to be kind, courteous, and friendly, whereas rude, impatient, and indifferent providers became the most common source of people’s complaints. The word “rude” was mentioned in 15.82% (429/2712) of the posts complaining about health care professionals’ attitude.

### Access to Care

A total of 24.4% (978/4008) of the posts mentioned the availability of an appointment for some professionals or the time spent waiting for an appointment or visit in the outpatient department. Among these posts, most complained about the long waiting times.

### Communication, Information, and Education

A total of 22.18% (889/4008) of the posts mentioned communication, information, and education they received about their clinical status, progress, prognosis, and processes of care. As health needs improve, people are no longer satisfied with receiving health care passively; instead, they expect to be involved in their treatment and care. When health care providers ignore their information needs, people report negative feelings and low satisfaction levels.

### Technical Competence

There were 393 posts (9.81%) describing providers’ technical competence, which referred mainly to venipuncture procedures. More than half of these posts (206/393, 52.4%) reported that nurses had excellent or poor invasive venipuncture skills or muscle injection skills.

### Efficacy of Treatment

A better health outcome is the primary intention of visiting hospitals; therefore, in addition to the service behaviors mentioned above, efficacy is also an important discussion topic. A total of 332 posts (8.28%) described satisfactory or unsatisfactory treatment efficacy.

## Discussion

### Principal Findings

Understanding patient experience is fundamental to providing patient-centred care, and social media platforms provide an additional source of information to complement traditional survey methods. This study revealed that people tend to release negative posts on social media platforms, which could attract more responses and trigger more discussions. The results of topic modeling highlight the interpersonal and functional aspects of care, especially the interpersonal aspects, which are often the “moment of truth” during a service encounter in which patients make a critical evaluation of hospital services.

This time-based content analysis using Weibo data revealed that social media platforms are gaining popularity for communicating and sharing hospital experience among the Chinese public. In this study, the frequency of patient experience–related posts significantly increased over time. From 2013‐2018, the average number of valid posts was less than 100 per year. However, after 2019, the average number of valid posts was nearly 1000 per year. Owing to the accessibility and authenticity of free-text online data, summarizing and analyzing the public’s disclosure on social media platforms can generate deeper and new insight into patient experience and serve as an important opportunity to identify areas for quality improvement action. Health care facilities need to recognize and seize this opportunity. At the policy level, social media has been viewed as an important avenue for monitoring the performance of health care providers. For example, the England NHS actively monitors social media to better understand and respond to patients’ voices [22]. However, few studies have monitored and mined unsolicited patient experience–related data on the internet. To our knowledge, this is the first study in China to understand patient experience using social media.

Many data-mining techniques have been used to understand free-text feedback regarding health care, such as LR, RF classifier, naïve Bayes, MNB classifier, support vector machine, and decision tree classifier [23,24]. The advantages of different methods vary. This study compared the performance metrics of each machine learning model and found that the LR, MNB, and RF had the highest precision and accuracy in predicting discussion topics related to patient experience; this finding is consistent with the findings of other studies [25]. This study therefore integrated LR, MNB, and RF to predict patient experience themes and sentiment, and the performance was higher than that of any single machine learning model [11,26]. The accuracy, precision, recall, and F-measure values in this study are all greater than 0.8. Accuracy reflects overall correctness, precision evaluates positive prediction quality, recall assesses sensitivity to positive instances, and the F-measure balances precision and recall. Our results indicate that the machine learning classification approach could effectively label free text and automatically classify patients’ views into one or more topic categories from given examples. Hospital administrators and clinicians can gain deep insight into patient experience via a machine learning approach.

Among the public discourse included in this content analysis, a wide range of patient experience themes were discussed. These themes can be divided into interpersonal and functional aspects. Interpersonal aspects include health care professionals’ attitude, emotional support, information provision, privacy protection, involvement of family members, and responding requests, which constitute the highest proportion of patient experience topics. Larson et al [27] also reported that patient experience mainly reflects the interpersonal aspects of health care services. This is especially true for provider attitude, which has been universally discussed and demonstrated as the critical attribute of patient experience and satisfaction in previous research [26,28]. This study found that themes associated with positive and negative emotional feedback both focus on the health care professionals’ attitude (eg, “rude” and “friendly”); the rude behavior experienced by patients may trigger dissatisfaction, while friendly behavior can receive praise. Maramba et al [29] conducted a textual analysis of free-text comments from patients and reported that “rude” was significantly associated with a worse experience. The functional aspects include access to care, technical competence*,* efficacy of treatment, physical comfort, error in treatment, fairness of care, and coordination of care. Similar findings were found in previous studies [30]. Many topics frequently appeared in the traditional hospital-initiated surveys (eg, access to care and physical comfort). There were also some topics that were not typically addressed, such as technical competence*,* efficacy of treatment, error in treatment, fairness of care and coordination of care, indicating that health care organizations can use social media platforms to identify unexpected patient experience aspects that may not be viewed by hospitals [31].

As shown in Table 3, the theme distribution of the whole sample and sentiments indicate that the interpersonal aspects of health care services play an important role in shaping a good patient experience. However, in most cases, when health care organizations develop quality improvement activities, they always focus on functional aspects, such as access to care, technical competence, efficacy of treatment, and physical comfort. The authentic voices of patients on social media offer a precious opportunity for health care organizations to reshape their services. Therefore, the use of the public’s comments on social media platforms will eventually lead to a more patient-centred health care system that will improve on interpersonal aspects of care.

This study also revealed that negative sentiment posts represented the highest share of all posts. Previous research has also demonstrated that many health care-related tweets are negative [32-34], which may be partly explained by the fact that social media platforms often serve as outlets for individuals to voice complaints and negative experiences more readily than positive experiences. Compared with positive posts, negative posts tend to attract more responses and trigger more discussions [35], therefore having a greater impact on the health care system. The health care system should monitor the rise of negative voices regarding services.

### Limitations

The Weibo platform provides an online public sphere in which people can express and discuss their authentic opinions. However, despite its widespread use, Weibo users do not represent the Chinese population; younger and higher socioeconomic populations are the main user groups on Weibo. Therefore, future studies could explore patient experience using other social media platforms, such as Douyin (Beijing Weibo Vision Technology Co, Ltd), which may attract more older adult individuals. Additionally, dissatisfied patients are more likely to voice complaints and their negative experiences than satisfied patients. Therefore, a selection bias exists, and other sources of complementary data, such as patients’ free-text comments in surveys and semistructured interviews, are necessary to further understand patient experience. However, owing to the benefits of accessibility, flexibility, and anonymity, social media has become a ubiquitous tool that allows individuals to openly gather, communicate, and share real-time information and feedback about their interactions with the health care services. We thus suggest that this research provides a starting point for Chinese hospital administrators and clinicians in terms of how social media analysis can improve health care.

### Conclusions

With the application of a mixed methods approach involving literature review, human annotation, and machine learning–based models, monitoring public disclosure about their interactions with hospitals on social media platforms can help to better understand gaps and potential opportunities for improving health care quality, serving as a supplemental source of data about patient experience. People tend to release negative posts on social media platforms, which could attract more responses and trigger more discussions. A wide range of patient experience themes were discussed, and the five most frequently discussed topics were “health care professionals’ attitude,” “access to care,” “communication, information, and education,” “technical competence,” and “efficacy of treatment,” highlighting the interpersonal and functional aspects of care.

### Implication

Social media has become common place and allows individuals to openly gather, communicate, and share real-time information and feedback about their interactions with health care services. Hospital administrators and clinicians should consider the value of social media and pay attention to what patients and their family members are communicating on social media. To increase the utility of this information, a machine learning algorithm can be used for topic modeling of patients’ views about health care providers and their care.

## Supplementary material

10.2196/59249 Multimedia Appendix 1 Patient experience theme framework.

10.2196/59249 Multimedia Appendix 2 Patient experience–related posts.

## Abbreviations

LR: logistic regression
MNB: multinomial naïve Bayes
NHS: National Health Service
RF: random forest

## Multimedia Appendix 1

Final coding framework.
