# Supplementary material for: Analyzing Patient Experience on Weibo: Machine Learning Approach to Topic Modeling and Sentiment Analysis
Source: JMIR Med Inform. 2024 Nov 29;12:e59249. doi: 10.2196/59249 (PMC11623958; doi:10.2196/59249)
Supplement: Multimedia Appendix 1 [file medinform-v12-e59249-s001.docx]

**Supplementary material 1 Patient experience theme framework**

| **Patient experience theme** | **Connotation** |
| --- | --- |
| Heath care professionals’ attitude | The manner that health care professionals interact with the customer during service encounters. |
| Access to care | The time spent waiting for admission or time between admission and placement in a room in an in-patient setting, and waiting time for an appointment or visit in the out-patient department. |
| Information, communication and education | Information, communication, and education on clinical status, progress, prognosis, and processes of care in order to facilitate autonomy, self-care and health promotion. |
| Technical competence | The technical skills of healthcare professionals, together with their adherence to high standard. |
| Efficacy of treatment | Outcomes in improving or maintaining health status. |
| Responding request | Healthcare professionals’ response to patients’ and their caregivers’ need. |
| Medical cost | Money spent on the medical service. |
| Physical comfort | Pain and other symptom experienced by patients. |
| Hospital environment | Clean and comfortable surroundings. |
| Privacy | The protection of body during physical examination. |
| Emotional support | Alleviation of fear and anxiety about such issues as clinical status, prognosis, and the impact of illness on patients, their families and their finances |
| Equipment | The quality or quantity of equipment provided by the hospital. |
| Service process | The convenience, quickness and efficiency of organisational processes. |
| Continuity of care | Coordination and integration of care across the health care system. |
| Error in treatment | Mistakes or errors in treatment or medication administration. |
| Involvement of family members | Awareness and accommodation of family members’ needs. |
| Sense of responsibility of staff | The conscience of being responsible for patients and work. |
| Excessive treatment | Unnecessary examination, drugs and medical procedures perceived by patients. |
| Food | The meal provided for patients by the hospital. |
| Fairness of care | The order of medical service or patients’ right of receiving in-time treatment. |
