# Supplementary figures and images for: Analyzing Patient Experience on Weibo: Machine Learning Approach to Topic Modeling and Sentiment Analysis
Source: JMIR Med Inform. 2024 Nov 29;12:e59249. doi: 10.2196/59249 (PMC11623958; doi:10.2196/59249)

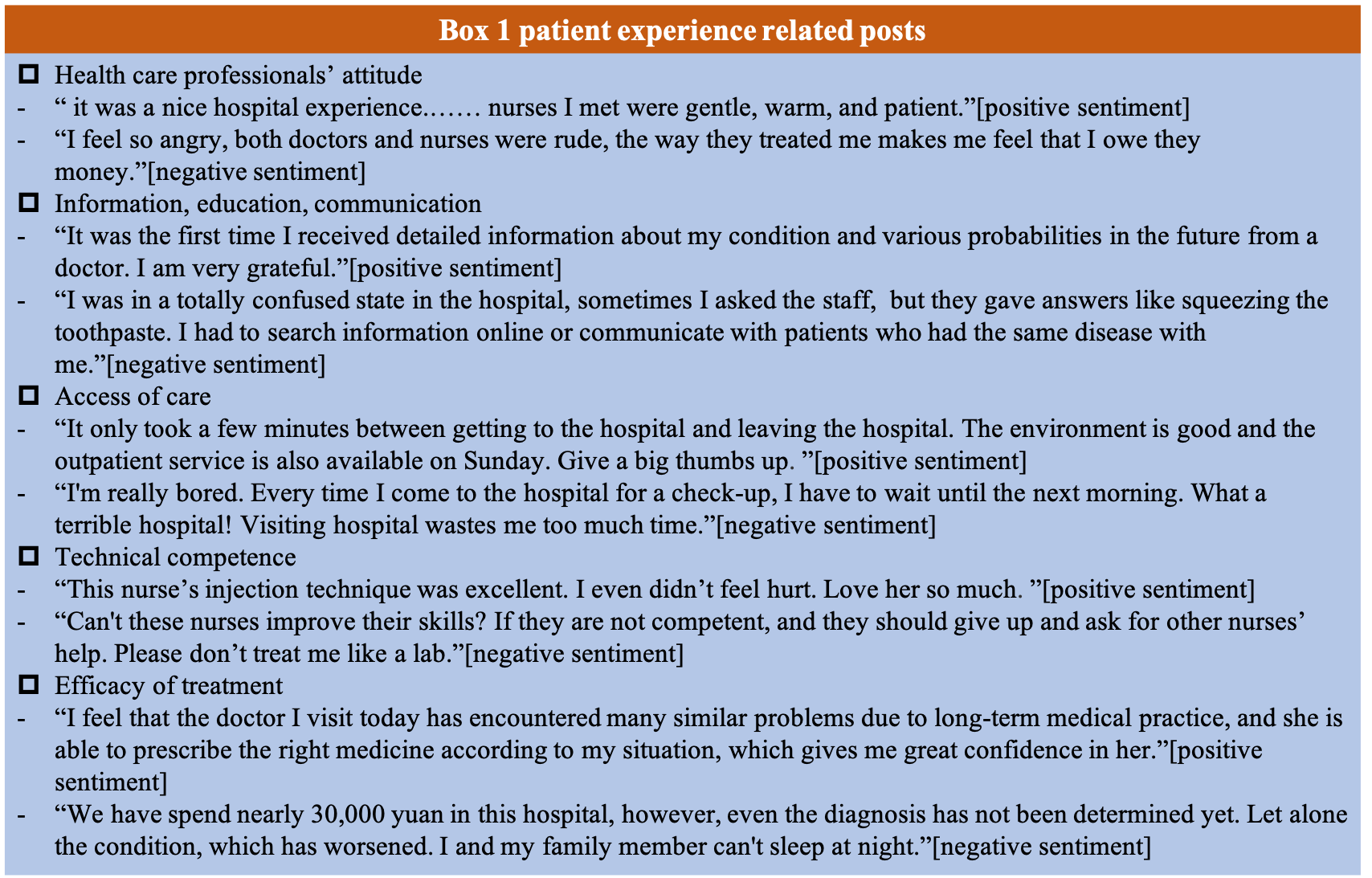

Supplement: Multimedia Appendix 2 [file medinform-v12-e59249-s002.png]
